# Genome‐wide analysis reveals conserved transcriptional responses downstream of resting potential change in *Xenopus* embryos, axolotl regeneration, and human mesenchymal cell differentiation

**DOI:** 10.1002/reg2.48

**Published:** 2015-11-26

**Authors:** Vaibhav P. Pai, Christopher J. Martyniuk, Karen Echeverri, Sarah Sundelacruz, David L. Kaplan, Michael Levin

**Affiliations:** ^1^ Biology Department and Center for Regenerative and Developmental BiologyTufts UniversityMedfordMassachusetts02155USA; ^2^ Center for Environmental and Human Toxicology and Department of Physiological SciencesUF Genetics Institute, University of FloridaGainesvilleFlorida32611USA; ^3^ Department of Genetics, Cell Biology and DevelopmentUniversity of MinnesotaMinneapolisMinnesota55455USA; ^4^ Department of Biomedical EngineeringTufts UniversityMedfordMassachusetts02155USA

**Keywords:** Axolotl, depolarization, differentiation, embryogenesis, ion channel, mesenchymal stem cells, microarray, transcriptome, *V*_mem_, *Xenopus*

## Abstract

Endogenous bioelectric signaling via changes in cellular resting potential (*V*_mem_) is a key regulator of patterning during regeneration and embryogenesis in numerous model systems. Depolarization of *V*_mem_ has been functionally implicated in dedifferentiation, tumorigenesis, anatomical re‐specification, and appendage regeneration. However, no unbiased analyses have been performed to understand genome‐wide transcriptional responses to *V*_mem_ change in vivo. Moreover, it is unknown which genes or gene networks represent conserved targets of bioelectrical signaling across different patterning contexts and species. Here, we use microarray analysis to comparatively analyze transcriptional responses to *V*_mem_ depolarization. We compare the response of the transcriptome during embryogenesis (*Xenopus* development), regeneration (axolotl regeneration), and stem cell differentiation (human mesenchymal stem cells in culture) to identify common networks across model species that are associated with depolarization. Both subnetwork enrichment and PANTHER analyses identified a number of key genetic modules as targets of *V*_mem_ change, and also revealed important (well‐conserved) commonalities in bioelectric signal transduction, despite highly diverse experimental contexts and species. Depolarization regulates specific transcriptional networks across all three germ layers (ectoderm, mesoderm, and endoderm) such as cell differentiation and apoptosis, and this information will be used for developing mechanistic models of bioelectric regulation of patterning. Moreover, our analysis reveals that *V*_mem_ change regulates transcripts related to important disease pathways such as cancer and neurodegeneration, which may represent novel targets for emerging electroceutical therapies.

## Introduction

Along with biochemical signals, cell behaviors such as migration, morphological change, proliferation, and differentiation are regulated by physical properties. In particular, bioelectric signaling among non‐neural cells has recently been shown to be an instructive component of pattern formation during regeneration, development, and cancer (Borgens 1988; McCaig et al. 2005; Zhao et al. 2006; Forrester et al. 2007; Stewart et al. 2007; Zuberi et al. 2008; Chernet & Levin 2013a; Yang & Brackenbury 2013). One important aspect of endogenous bioelectricity is resting potential or *V*_mem_ (Levin 2014b). *V*_mem_ controls proliferation, differentiation, apoptosis, and migration of a wide range of cell types (Gilbert & Knox 1997; Wang et al. 1999; Weihua et al. 2005; Blackiston et al. 2009; Sundelacruz et al. 2009), including mammalian stem cells (Sundelacruz et al. 2008, 2013a; Pillozzi & Becchetti 2012; Swayne & Wicki‐Stordeur 2012; Shen et al. 2013; Sundelacruz et al. 2013b; Wang et al. 2014). Moreover, spatio‐temporal patterns of *V*_mem_ have been shown to specifically regulate growth and form (Levin 2012, 2013, 2014a; Adams & Levin 2013; Tseng & Levin 2013a). Ion channel activity, and the resulting voltage gradients, regulate shape, size, and positional information of organs in *Drosophila*, planaria, fish, frog, salamander, and mouse (Adams et al. 2007; Ozkucur et al. 2010; Lange et al. 2011; Dahal et al. 2012; Beane et al. 2013; Perathoner et al. 2014), as well as being responsible for several classes of developmental malformation in humans (Galanopoulou 2010; Tristani‐Firouzi & Etheridge 2010; Masotti et al. 2015). Bioelectric signals can trigger the formation of whole ectopic organs, such as in the case of eye development (Pai et al. 2012a), and can stimulate the repair of complex tissues such as spinal cord (Borgens et al. 1990, 1999; Shapiro et al. 2005; Tseng et al. 2010). Endogenous bioelectric gradients can also control the morphogenesis of limbs, faces, and whole body axes (Tristani‐Firouzi & Etheridge 2010; Marrus et al. 2011; Vandenberg et al. 2011), making these gradients an important modality for targeted intervention in regeneration and bioengineering applications (Levin & Stevenson 2012).

Despite the extensive functional data summarized above, there remains limited insight into specific pathways and mechanisms that directly link *V*_mem_ to such phenotypic changes. Recent work has identified several transduction mechanisms by which voltage potential change is converted into chromatin modification and changes of expression of a handful of specific target genes (Levin 2012; Tseng & Levin 2012) in several contexts. However, no systematic analysis has been performed to identify and integrate genome‐wide transcriptional changes following steady‐state depolarization in vivo, or to suggest novel biomedical endpoints for *V*_mem_ modulation. Moreover, while bioelectricity has been investigated in many phyla, the evolutionary conservation of downstream signaling pathways has not been fully described. For example, does the bioelectric code (the mapping of voltage states to specific organ outcomes) function via the same transcriptional programs in diverse organism morphologies, or is there a significant divergence in the downstream responses to bioelectrical signaling? Answering such questions is crucial, not only to fully understand the developmental role of physical forces, but also to help establish roadmaps for human regenerative medicine from data on bioelectric control of patterning in model systems of development and repair. Such roadmaps would also provide a path forward for the systematic probing of the pathways and the control achievable via *V*_mem_, analogous to what has been pursued for biochemical signaling mechanisms over the past few decades.

Here, we use genome‐wide analysis of transcriptomes as a tool for gaining insight into transcriptional cascades, and the degree to which these are conserved, downstream of *V*_mem_ change. Such strategies have been applied in several model organisms (Altmann et al. 2001; Baldessari et al. 2005; Chalmers et al. 2005; Tomancak et al. 2007; Yanai et al. 2011) and assist in acquiring a holistic view with systematic characterization of how gene expression is regulated towards functional paths during processes such as development or reproduction (Martyniuk & Denslow 2012; Langlois & Martyniuk 2013). In this study, we analyzed the effects of specific depolarization events on transcriptional profiles in *Xenopus laevis* embryos during development. We then compared these data with those from the regeneration of spinal cord in axolotl, *Ambystoma mexicanum*, and human mesenchymal stem cells (hMSCs). The strategy was to identify common features of signaling downstream of *V*_mem_ change along two orthogonal dimensions: species (frog, salamander, and human), and cellular context (embryogenesis, regeneration, and stem cell differentiation in vitro).

## Results

### Microarray analysis of depolarized model systems

Regulation of resting potential (*V*_mem_) of a wide variety of cell types plays an important role in embryogenesis, regenerative response, and cancer (Borgens et al. 1977b; McCaig et al. 2005; Blackiston et al. 2009; Pullar 2011; Lobikin et al. 2012; Adams & Levin 2013; Chernet & Levin 2013b, 2014; Tseng & Levin 2013b; Levin 2014a; Pai & Levin 2014). In order to understand the role of bioelectricity in pattern formation, and to harness this signaling modality for biomedicine, it is important to understand the transcriptional networks downstream of specific *V*_mem_ change. The microarray experiments reported here (from the three different species *Xenopus laevis*, axolotl and human) represent single time point experiments, performed to enable direct comparison of multiple transcriptomics datasets. In the following discussion we focus on only some of the biological themes affected by depolarization; our decision was guided by our research interests and paper‐length limitations. However, all the data for processes affected by depolarization are provided in the Appendices, to enable others to pursue any other interesting leads.

#### Xenopus

A recent study of transcriptional changes during *Xenopus* embryonic development identified dynamic changes in membrane hyperpolarization gene networks which were decreased in early development but increased later in development at stage 34 (Langlois & Martyniuk 2013). Here, we used microarray analysis to identify transcripts that are regulated specifically by depolarization (induced by the activity of each of two very different depolarizing channels, as is done during bioelectric induction of patterning changes in vivo). We focused on a single time point, just after mid‐gastrula transition, when new transcription begins (Woodland & Gurdon 1968; Forbes et al. 1983; Cascio & Gurdon 1987). Further detailed studies with different time points during development will be explored in future work, to understand long‐term, temporal aspects of *V*_mem_ regulation of gene regulatory networks during embryonic development.

**Figure 1:**
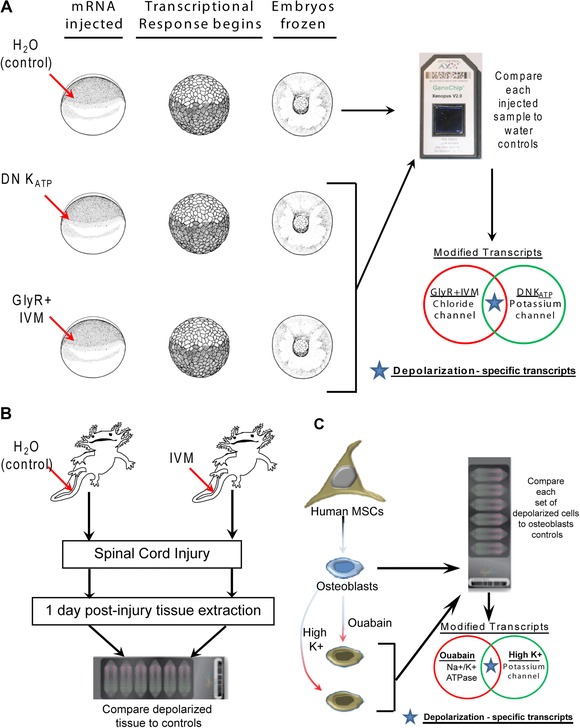
(A) Experimental design for the *Xenopus* microarray experiment. *Xenopus* embryos were microinjected at the one‐cell stage with water (control) or mRNA for dominant‐negative K_ATP_ (DN K_ATP_, 666 construct) or GlyR channel mRNA. GlyR‐injected embryos were incubated in channel opener drug ivermectin (IVM). The transcriptional response begins in the *Xenopus* embryos at stage 8. The embryos were flash‐frozen at stage 11, mRNA was extracted, and transcripts were compared between the experimental and control samples. Extracts from 50 embryos was pooled for each experimental group. Only those transcripts that were similarly modified in both the GlyR+IVM and DN K_ATP_ groups were used as depolarization‐specific modified transcripts. (B) Experimental design for the axolotl microarray experiment; 2−3 cm axolotl were used. The central canal of the spinal cord was pressure injected with vehicle (water, controls) or injected with IVM (depolarization). Immediately after injection spinal cord injury was performed by removing a small portion of the cord. One day post‐injury the area of injury was removed after anesthetizing the animals. Tissues from 10 animals were pooled for each experiment. Extracted RNA was used to detect changed transcripts in IVM‐injected animals in comparison to control (water injected). (C) Experimental design for the primary human mesenchymal stem cells (hMSCs) microarray experiment. hMSCs were induced to differentiate into osteoblasts. These osteoblasts were then treated with ouabain (Na^+^/K^+^ ATPase inhibitor) or incubated in medium with high potassium (both depolarizing conditions). The mRNA extracted from the treated cells was compared with that of untreated osteoblasts (controls). Among the modified transcripts only those that were similarly modified in both the treatments were used as depolarization‐specific modified transcripts.

Changes in endogenous *V*_mem_ patterns encode important signals for individual cells and also for large‐scale patterning programs. To begin to analyze the global transcriptional targets of such change, we induced specific depolarization in frog embryo cells by misexpression of a depolarizing ion channel. We previously showed that this technique results in a specific, coherent anatomical change (induction of well‐formed ectopic eyes throughout the animal [Pai et al. 2012a]), and it is a strategy routinely used to investigate bioelectric signaling (Adams & Levin 2013; Adams et al. 2013). *Xenopus laevis* embryos were injected with either 666 (DN‐K_ATP_) (Hough et al. 2000), glycine‐gated chloride channel (GlyR) (Davies et al. 2003), or water (controls). The mRNA extracted from each of these treatments (*n* = 50 each) was used for microarray analysis using an Affymetrix *X. laevis* Genome Genechip 2.0 Array (Fig. 1). DN‐K_ATP_ has been previously shown to cause depolarization of the injected cells in *Xenopus* embryos by inhibiting K_ATP_ channels (Hough et al. 2000; Pai et al. 2012a). Similarly, expression of the GlyR channel in the presence of the channel opener drug ivermectin (IVM) also depolarizes the injected cells in *Xenopus* embryos (Davies et al. 2003; Blackiston et al. 2011; Pai et al. 2012a). We used two different (K^+^ and Cl^−^ ion flux) channels that both depolarize embryonic cells, in order to focus on genes whose transcription is specifically responsive to depolarization, not sodium or potassium signaling per se (nor on any possible ion‐independent functions of one channel protein).

In the *Xenopus* dataset, 380 genes were significantly upregulated in both experimental groups (DN‐K_ATP_ and GlyR+IVM), in comparison to controls (water‐injected), with at least a twofold increase in gene expression (Appendix S1). There were 140 genes that were commonly downregulated in both experimental groups in comparison to controls, with at least a twofold decrease in gene expression (Appendix S1). Since the list of downregulated genes was small, we focused our analysis on the significantly upregulated gene list from the *Xenopus* dataset. To organize our data into functional categories, we used subnetwork enrichment analysis (SNEA). These networks were then verified with the results of a functional classification analysis using the PANTHER database.

#### Axolotl

To begin to understand the degree of conservation of these transcriptional responses, we also performed a microarray analysis of axolotl spinal cord regeneration. Control (vehicle water) treated animals were compared with animals treated with the depolarizer IVM (opens endogenous GlyR channels leading to Cl^−^ efflux). In these experiments, the IVM or control vehicle (water) was directly injected into the central canal of the axolotl spinal cord, and the spinal cord was injured by removing a portion of the spinal cord (Diaz Quiroz & Echeverri 2012; Sabin et al. in review) (Appendix S1). Tissue samples were harvested 1 day post‐injury, and tissue from 10 axolotls was pooled and RNA extracted for microarrays. Arrays were carried out using a commercially available custom Affymetrix axolotl array. In the axolotl dataset, 756 genes were significantly upregulated in the experimental groups (IVM treatment 1 day post‐injury), in comparison to controls (water‐injected), with at least a twofold increase in gene expression (Appendix S1). There were 753 genes that were commonly downregulated in the experimental group in comparison to controls, with at least a twofold decrease in gene expression (Appendix S1). We did not restrict the bioinformatics analysis by fold change, and have included all genes that showed an uncorrected *P* < 0.05 (Appendix S1).

#### Human mesenchymal stem cells (hMSCs)

To extend the analysis to human stem cells, as well as to compare the above in vivo assays with results obtained in vitro, we compared the above microarray data to microarray analysis of osteoblasts derived from undifferentiated hMSCs. Normal osteoblast differentiation medium has low extracellular K^+^. The osteoblasts were depolarized by (1) elevating extracellular K^+^ via addition of potassium gluconate (40 mmol/L) into the medium (elevating the extracellular K^+^ reverses the electrochemical gradient for K^+^ leading to depolarization of cells) or (2) treatment with the Na^+^/K^+^ ATPase inhibitor ouabain (OB) (10 nmol/L) in the medium. Depolarization induced by these concentrations of OB and K^+^ has been confirmed using voltage‐sensitive dyes and/or sharp intracellular recordings (Sundelacruz et al. 2008; and Kaplan DL et al., unpublished data). Osteoblasts in the normal differentiating medium were used as controls (Sundelacruz et al. 2013a). The microarray was performed using Illumina Human WG6 v3 Expression BeadChip arrays, which have 48,804 probe sets, of which over 27,000 represent coding transcripts with well‐established annotation. We used two different treatment conditions that both depolarize cells, in order to focus on genes whose transcription is specifically responsive to depolarized groups, *n* = 3, and a non‐depolarized osteogenic control group, *n* = 3, were used. In the human dataset, 2777 genes were significantly upregulated in both the experimental groups (high K^+^ in medium and OB treatment), in comparison to controls (normal differentiation medium) (Appendix S1). There were 2706 genes that were downregulated in comparison to controls (Appendix S1). We however did not restrict the bioinformatics analysis by fold change, and have included all genes that showed a *P* < 0.01 (Appendix S1).

In summary, we reasoned that common pathways identified in the comparison (across species and across process type) represent those cell signaling pathways and processes most probably affected following membrane depolarization. However, additional efforts must continue to verify the role of changing *V*_mem_ on these pathways.

### Subnetwork enrichment analysis (SNEA) of depolarization transcripts from all three datasets

**Figure 2:**
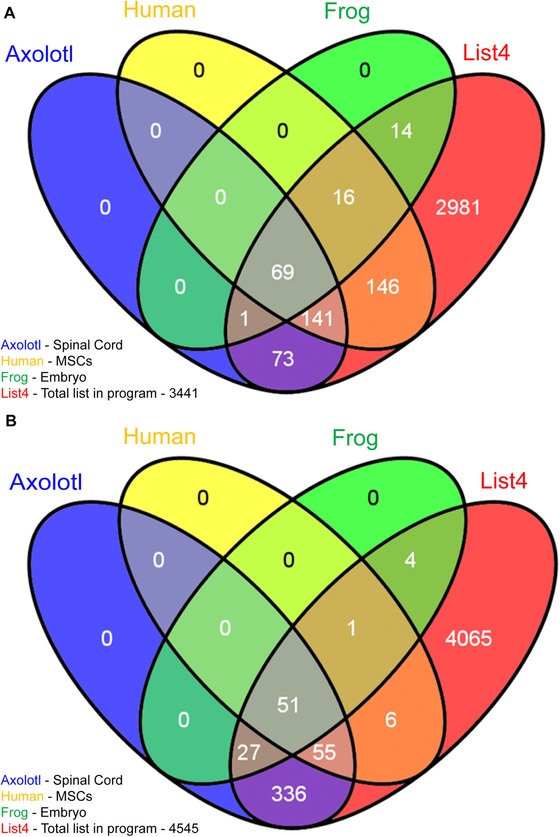
(A) Cell processes affected in each dataset. List 4 is the total list of all cell processes in the program. There were 2990 cell processes that were not affected in any of the three datasets. (B) Diseases affected in each dataset. List 4 is the total list of all diseases in the program. There were 4065 diseases that were not affected in any of the three datasets.

**Figure 3:**
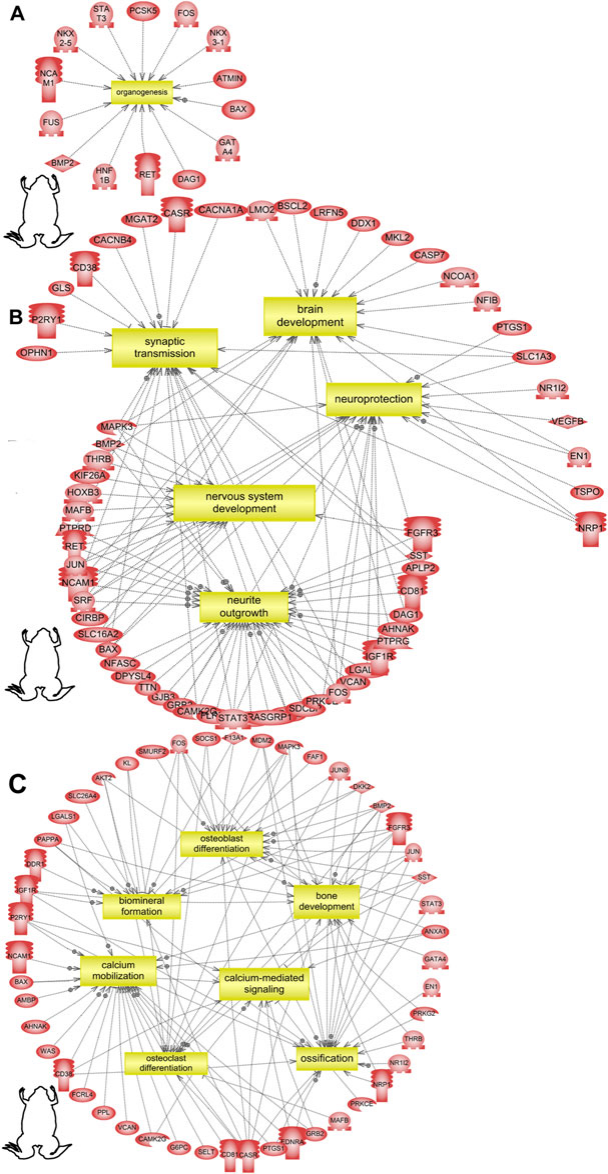
Subnetwork enrichment analysis of the *Xenopus* dataset identified (A) organogenesis as a process significantly enriched with a list of key genes that are involved in organogenesis indicated in red, (B) regulated genes that are involved in brain/neural development, (C) regulated genes that are involved in bone morphogenesis. Acronyms can be found in Appendix S5. Gene functions can be found in Table S1.

**Figure 4:**
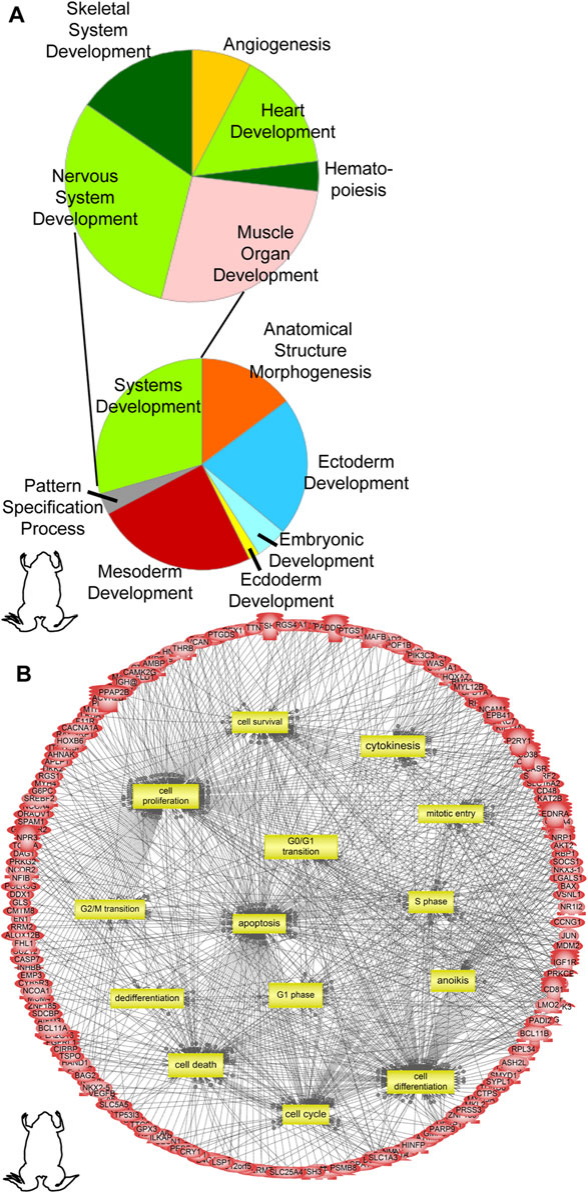
(A) Pie chart of the functional classification of the *Xenopus* dataset using the PANTHER database showing enrichment of embryonic developmental processes particularly organ development. (B) Subnetwork enrichment analysis of the *Xenopus* dataset identified regulated genes involved in “large‐scale functions” (those seen across germ layers and various tissues during development) like cell cycle, differentiation, dedifferentiation, proliferation, etc. Acronyms can be found in Appendix S4. The gene list and their fold change can be found in Appendix S1.

**Figure 5:**
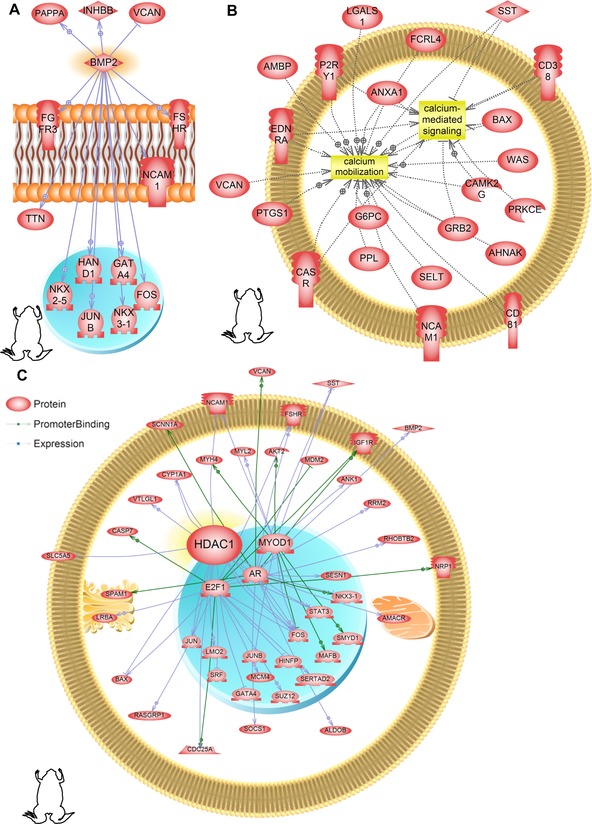
Subnetwork enrichment analysis of the *Xenopus* dataset identifies regulated genes that are involved in (A) BMP2 signaling, (B) calcium signaling, and (C) histone deacetylase (HDAC) signaling. Acronyms can be found in Appendix S5.

**Figure 6:**
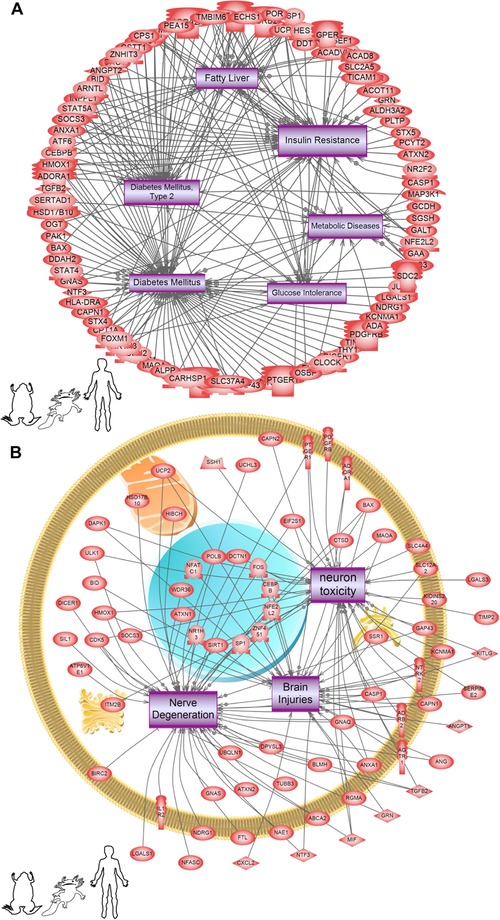
Subnetwork enrichment analysis (SNEA) of all three (frog, axolotl, and human) datasets identifies regulated cell processes and diseases common in all three datasets that are involved in metabolic disease pathways. The pathways depicted are the ones mapped with axolotl genes, but these same processes and diseases were affected in both frog and human (i.e., the processes were common in all three). The specific genes from the frog and human datasets may be different but they affect the same cell processes and diseases. (A) metabolic disease pathways, and (B) brain‐related pathways within the “disease” database. Acronyms can be found in Appendix.

SNEA was performed for each of the three datasets, an analysis that determines if there are significantly enriched processes in a list (based on gene function) compared to a background list (annotated subnetworks in the database based on mammalian literature) (Figs. 2−6, S1−S3, Tables 1, 2, S1 and S2). Common subnetworks were identified, and this was followed by manual grouping into major biological themes.

**Table 1:** Cell process subnetworks that are common between all three (frog, axolotl, and human) datasets. Differentially expressed genes in each dataset are statistically more likely to be involved in these processes. Total hits refers to total number of genes in the category in the database.

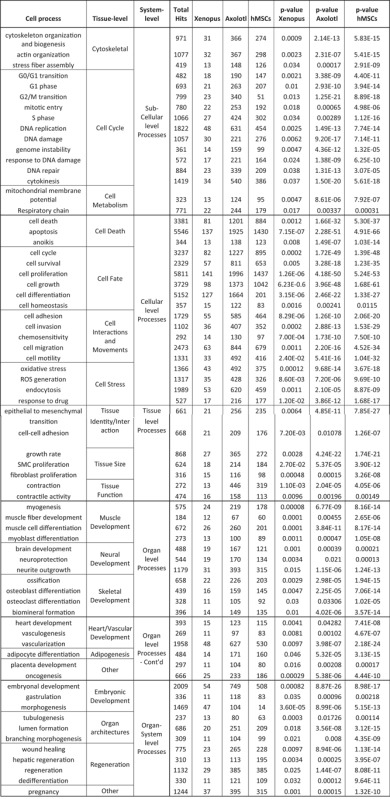	

ROS, reactive oxygen species; SMC, smooth muscle cell.

**Table 2:** Disease subnetworks that are common among all three (frog, axolotl, and human) datasets. Differentially expressed genes in each dataset are statistically more likely to be involved in these processes. Total hits refers to total number of genes in the category in the database.

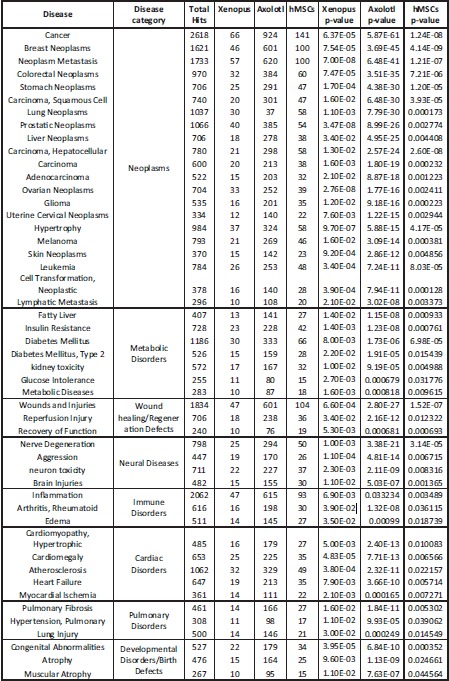	

Out of a total 3441 cell processes in the database (Fig. 2A, List 4), there were 100, 284, and 372 cell processes affected in *Xenopus*, axolotl, and human cells respectively following depolarization (Fig. 2A). There were 2981 cell processes unaffected by any treatments across all three experiments (Fig. 2A, red). Similarly, out of a total 4545 disease networks in the database (Fig. 2B, List 4), there were 83, 469, and 113 diseases affected in *Xenopus*, axolotl, and human cells respectively following depolarization (Fig. 2B). There were 4065 disease networks unaffected by any treatments across all three experiments (Fig. 2B, red). It is interesting to note that a total of 69 cell processes and 51 disease networks were enriched in common between the frog, axolotl and human datasets in response to depolarization (Fig. 2A, B and Tables 1 and 3). Each of these networks contained a minimum of 10 entities/genes. Comparing these to the total list of possible cell processes and disease networks (Fig. 2A, B) revealed that only a subset of cell processes and disease networks were affected by depolarization at the level of the transcript.

**Table 3:** List of cell signaling pathways from panther.org analysis of the *Xenopus* dataset.

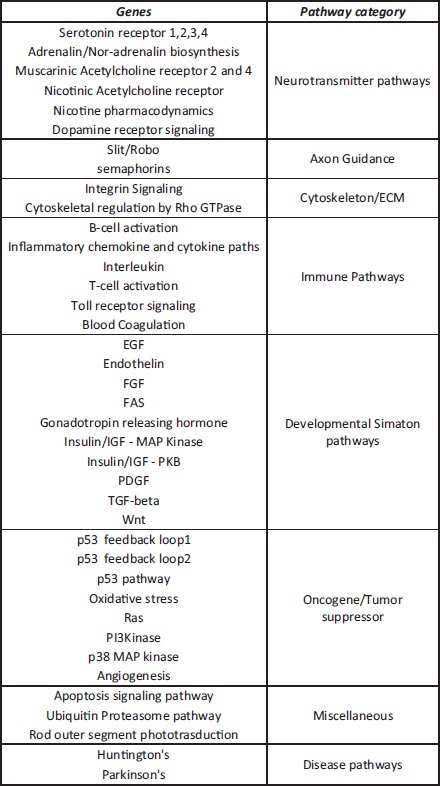	

Of the common processes, there was approximately 25% that were related to organ development (17/69) while 23% were related to cell cycle and cell function (16/69). All subnetworks are presented in Appendix S2. Some examples of interesting depolarization‐affected gene networks involved in organ development are depicted in Figure 3. Noteworthy is that the gene networks regulated by depolarization include organ/organ systems belonging to all three embryonic germ layers (ectodermal, mesodermal, and endodermal) (Figs. 3, S1 and Table S1). This was found to be the case in the other two (axolotl and human) datasets as well (Table 1). Transcripts involved in brain/neural development (Fig. 3B), skeletal/bone development (Fig. 3C), immune system development (Fig. S1), and adipose development (Fig. S1) were found to be significantly affected by depolarization.

*V*_mem_ is known to regulate cell behaviors, although the transcriptional mediators of voltage‐regulated shape change are poorly understood (Ghiani et al. 1999; Bauer & Schwarz 2001; Chittajallu et al. 2002; Sundelacruz et al. 2008; Blackiston et al. 2009; Becchetti & Arcangeli 2010; Schwab et al. 2012; Stock et al. 2013). To address this, we searched for “large‐scale functions” (regulated processes that are found across all tissues and organ structure and are necessary for proper developmental morphogenesis and anatomical sculpting) which may be regulated by depolarization. We identified several large‐scale function gene clusters within the frog dataset (Fig. 4B, Table 1). Depolarization‐mediated regulation of such large‐scale functions was also found to be a common theme across all three (frog, axolotl, and human) datasets (Table 1). Our analysis confirmed *V*_mem_ regulation of key cell cycle events in vivo during development (G_2_/M transition, mitotic entry, S‐phase and DNA replication) (Table 1 and Fig. 4). Our analysis identified putative transcriptional targets by which *V*_mem_ regulates apoptosis (Table 1 and Fig. 4), and also identified other cell death mechanisms like anoikis that are regulated by *V*_mem_ (Table 1). Our analysis also identified, for the first time, fate specification genes regulated by *V*_mem_ for tissues from all three germ layers (Table 1) (e.g., *nkx2.5*, *gata4*, *nkx3.1*, *hnf1b*) (Tronche & Yaniv 1992; Shiojima et al. 1995; Bhatia‐Gaur et al. 1999; Zhu et al. 2000; Kohler et al. 2008; Li et al. 2012; Zhou et al. 2012).

We were interested in identifying cellular‐level signaling pathways that are responsive to depolarization, particularly those that are conserved across species. SNEA was performed for “expression targets” in Pathway Studio. Indeed, a common subset was found to be differentially affected in all three datasets (Table S2). During embryonic development a collection of conserved juxtacrine and paracrine signals (consisting of growth factors, morphogens, hormones, and cytokines) are known to integrate together in various spatial and temporal permutations−combinations to drive specific developmental patterns collectively defined here for the first time as a “developmental simaton”. The majority of the common subset of pathways identified in our experiments belonged to the developmental simaton (Table S2). These include IGF, FGF, BMP/TGFβ, HGF, EGF, PDGF, and gonadotropins among others. SNEA for “expression targets” revealed that BMP2 may be an important developmental simaton pathway regulated by depolarization (Figs. 5A, S2A, C and Appendix S3) due to a significant number of transcripts with altered expression in the datasets that were downstream of this signaling molecule in all three species. Depolarization also regulates cell cycle (oncogenic/tumor suppressor) signals across species (Table S2). Another set of depolarization‐regulated signals conserved across species (frog, axolotl, and human datasets) are ion translocators, particularly those mediating calcium and chloride transport and signaling (Figs. 5B, S2B, C). Some examples of depolarization‐regulated ion channel (particularly calcium and chloride) genes in the *Xenopus* dataset are voltage‐dependent calcium channel p/q type alpha 1A (*cacna1a*), voltage‐dependent calcium channel beta unit 4 (*cacnb4*) and pendrin (*slc26a4*).

To determine which diseases might be associated with depolarization, we used SNEA to query disease networks associated with differential gene expression in all three (frog, axolotl, and human) datasets. The largest group of diseases were neoplasms associated with various tissues (Table 2). Surprisingly, a significant number of metabolic disease networks like diabetes, insulin resistance, and glucose intolerance were also regulated by depolarization (Fig. 6A and Table 2) as were neural disorders (Figs. 6B and S3).

### Protein annotation through evolutionary relationship (PANTHER) analysis of depolarization transcripts from *Xenopus* dataset

In order to confirm the observations seen by SNEA, we performed an independent analysis of the frog dataset using the PANTHER (protein annotation through evolutionary relationships) functional classification system (Mi & Thomas 2009; Mi et al. 2013a) (Fig. 4A and Table 3). This algorithm is designed to classify genes and their proteins to specific functions. We used the “biological process” function, which clusters the genes and their functions in the context of larger networks. Since a gene can be classified according to more than one term, the pie chart is calculated according to the number of “hits” to the terms divided by the total number of “class hits.” A class hit indicates an independent (not parent or child to each other) ontology term (Mi & Thomas 2009; Mi et al. 2013a). Developmental process was one of the 13 biological processes enriched from the entire gene list of upregulated genes. PANTHER analysis also suggested that genes upregulated by depolarization in the frog dataset were involved in regulating embryonic developmental processes that spanned across all three germ layers (Fig. 4A), similar to the SNEA observations (Tables 1, S1 and Fig. 4). These processes included anatomical structure morphogenesis, pattern specification process, ectoderm development, endoderm development, mesoderm development, and systems development (Fig. 4A). A deeper analysis of genes clustered within the “systems development” category showed that the regulated genes were involved in the development of organ/organ systems, for example muscle development, nervous system development, skeletal system development, and heart development (Fig. 4A). These categories are in line with those obtained using SNEA (Tables 1, S1 and Fig. 3). The PANTHER analysis supports the observation that depolarization‐mediated changes in mRNA abundance affect organogenesis of tissues and organs across all three germ layers during embryonic development.

We independently analyzed the pathways in the frog dataset using the PANTHER database classification system (Mi & Thomas 2009; Mi et al. 2013a) (Table 3). Developmental simaton pathways and cell cycle regulatory (oncogenic/tumor suppressor) pathways were a major subset of the pathways regulated by depolarization similar to SNEA (Tables 3 and S2). Neural, neurological disorders and immune pathways were some other pathways that overlapped with the SNEA results (Tables 2, 3, Figs. 6, S1 and S3).

## Discussion

We investigated the evolutionary conservation of transcriptional responses to depolarization across two dimensions: species (comparing between frog, axolotl, and human), and process type (development, spinal cord regeneration, and stem cell differentiation). Our analysis included single time points, not a temporal time course. Comparative analysis for the effect of *V*_mem_ signals was done under similar *V*_mem_ conditions (depolarization). Thus, we reasoned that common pathways identified in the comparison represent those cell signaling pathways and processes most likely affected following membrane depolarization. However, additional efforts must continue to verify the role of changing *V*_mem_ on these pathways. Below we discuss a subset of genes and processes regulated by *V*_mem_ (selected by us based on the likely impact in several key fields of biology); a complete list is provided in Appendices S1−S4. We focused on *Xenopus* because it is in this system that the largest number of published mechanistic data are available, demonstrating that endogenous *V*_mem_ gradients and their modulation can be used to achieve predictable and coherent changes in body pattern formation. Hypotheses derived from our analysis will be most readily first tested in frog, but then moved into human tissues and other model systems. As such, the discussion remains focused on this species with reference to the other two models as a comparison.

### Depolarization‐mediated changes in gene expression patterns suggest that certain biological processes are highly sensitive

To identify cell processes and diseases related to genes affected by depolarization events, differentially expressed genes from all three datasets (*Xenopus*, axolotl, and human) were queried in Pathway Studios using SNEA. Comparing these to the total list of possible cell processes and disease networks (Fig. 2A, B) revealed that only a subset of cell processes (460 out of 3441, ∼13%) and disease networks (480 out of 4545, ∼10%) were affected by depolarization at the level of the transcript. The above data on large‐scale global changes in gene expression pattern reveal that the effect of membrane potential depolarization is not indiscriminate or random. While additional functional experiments are required, transcriptomics suggest that depolarization may affect specific cell processes and diseases (either directly or indirectly, via secondary genetic or biochemical signals), commonly across the three species. A total of 69 cell processes and 51 disease networks were enriched in common between the frog, axolotl, and human datasets in response to depolarization (Fig. 2A, B and Tables 1, 2), suggesting that these are the downstream events most probably affected by depolarization. The major biological theme underlying depolarization‐mediated changes in cell processes appears to be the regulation of tissue and organ development during embryogenesis (17/69, 25%, related to organ development, and 16/69, 23%, related to cell function and cell cycle) (Table 1 and Appendix S2). This includes depolarization‐mediated regulation of cellular level processes (cell fate, cell death, cell interactions), tissue level processes (tissue size, function, and interaction), organ level processes (development of muscle, neural, skeletal, and heart/vascular tissues), and organ‐system level processes (embryonic development processes, organ architectures, regeneration) (Table 1).

It is important to note that the number of cell processes affected is higher in the human dataset but the number of disease processes affected is higher in the axolotl dataset. This is probably due to the differences in the transcriptomes of specific cell types in culture (mesenchymal) versus an entire spinal cord region including the surrounding cells and tissues involved in regeneration (with contributions from all three germ layers). The disease pathways for the human dataset derived from a single germ layer and homogenized cell culture are limited compared to the axolotl dataset derived from all germ layers and numerous kinds of cells in that tissue. It is a conservative approach to analysis, as a much larger list of commonalities would be expected if a comparison were to be made with a human tissue sample similar to the axolotl sample with contributions from all three germ layers.

### Depolarization regulates organogenesis networks (nervous, skeletal/bone, adipose, and immune system) across all three germ layers

Organogenesis consists of complex series of spatial and temporal patterning events. Since our data analyzed transcriptional state at only one point, it is revealing only a snapshot of the responses to depolarization. However, previous studies analyzing *Xenopus* developmental processes at multiple time points show *V*_mem_ as a critical regulator of organogenesis at several stages (Langlois & Martyniuk 2013). To further understand what aspects of organogenesis are affected by depolarization, we analyzed the frog dataset for regulation of organ development processes using SNEA. Noteworthy is that the gene networks regulated by depolarization include organ/organ systems belonging to all three embryonic germ layers (Fig. 3 and Table S1). This was found to be the case in the other two (axolotl and human) datasets as well (Table 1).

*V*_mem_ coordinates differentiation and morphogenesis of ectodermal craniofacial (Vandenberg et al. 2011) and eye and brain structures in *X. laevis* embryogenesis (Pai et al. 2012a, 2012b, 2015). SNEA identified brain/neural tissue patterning as significantly regulated processes by *V*_mem_, supporting the previous observations. However, this analysis suggests that specific processes within brain/neural tissue patterning are regulated by *V*_mem_ signals (synaptic transmission, neuroprotection, neurite outgrowth), and identifies the genes regulated by *V*_mem_ in each of these processes (Fig. 3B and Table 1). Similar *V*_mem_‐mediated regulation of target genes involved in neural/brain tissue development and disease was also observed in axolotl spinal injury and human cells (Figs. 3B, 6B, S3 and Table 1). This analysis generates testable hypotheses for discovering the precise role and mechanism of *V*_mem_ in regulation of neural tissue patterning, especially with respect to bioelectrical control of the NCAM, BMP, FGF, serotonergic, adrenergic, and dopaminergic pathways.

In addition to ectodermal tissues (brain, eye, and craniofacial structures), this analysis for the first time suggests that *V*_mem_ also regulates mesodermal (heart, muscle, bone, adipose, immune system) and endodermal (kidney, pancreas, lung) organogenesis (Fig. 3 and Tables 1 and S1). Gene networks related to skeletal/bone development were identified (Fig. 3C) based on the differentially expressed transcripts. These included osteoclast differentiation, osteoblast differentiation, ossification, biomineralization, calcium mobilization, and calcium‐mediated signaling. Analogous to neural/brain development, the gene targets significantly enriched in skeletal/bone development subprocesses and the adipocyte differentiation process (Fig. S1) are found to overlap with similar gene‐target‐enriched subprocesses regulated by depolarization in axolotl and human datasets (Table 1). These observations are further supported by recent studies showing *V*_mem_‐mediated regulation of adipogenic and osteogenic differentiation of hMSCs (Sundelacruz et al. 2008, 2013a), and the induction of regeneration of bony appendages induced by bioelectric signaling (Borgens et al. 1977a, 1977b, 1979, 1983, 1984; Sisken et al. 1984; Tseng & Levin 2013a).

Endoderm mainly forms the tubular lining of internal organs associated with digestive tract (gut, liver, pancreas, and kidneys) and lungs (Fukamachi & Takayama 1980; Kedinger et al. 1990; Gilbert 2010). We found evidence of *V*_mem_ regulation of endodermal genes and processes: in *Xenopus*, the *hnf1b* gene is involved in kidney and pancreas development and *atmin* is involved in kidney and lung tubulogenesis (Table S1). Also *V*_mem_ regulation of endodermal processes such as gastrulation, tubulogenesis, lumen formation, branching morphogenesis, and hepatic regeneration (Gilbert 2010) was found to be a common significantly enriched gene target theme in all three organisms (Table 1). The limited observed effect of *V*_mem_ on transcripts involved in endoderm organogenesis could be a consequence of the temporal limitations of this study, since endoderm development programs were not highly active at the time point of sample collection.

PANTHER analysis of the frog dataset also showed that “developmental processes” was one of the 13 enriched biological processes and those subsets of genes were involved in regulating embryonic developmental processes that spanned across all three germ layers (Fig. 4A), similar to the SNEA observations (Tables 1, S1 and Fig. 4). Moreover, the genes were involved in muscle, skeletal, nervous, and cardiac/heart system development (Fig. 4A), categories in line with those obtained using SNEA (Tables 1, S1 and Fig. 3). The PANTHER and SNEA together indicate that depolarization‐mediated transcriptomic changes affect organogenesis of tissues and organs across all three germ layers during embryonic development.

### Depolarization employs basic large‐scale functions in its regulation of organogenesis networks across all three germ layers

Tissues and organ systems develop through regulation of basic large‐scale functions such as proliferation (size control), cell differentiation (fate determination), dedifferentiation (cancer and regeneration), and cell death (morphogenetic sculpting) (Abud 2004; Stanger 2008a, 2008b). These functions collectively direct changes in both growth and form to generate the appropriate anatomical pattern. Functional data have shown that bioelectric state change could reverse or duplicate whole body axes, including the head−tail axis in planaria (Oviedo et al. 2010; Beane et al. 2011) and the left−right axis in frog, chick, and zebrafish embryogenesis (Levin et al. 2002; Adams et al. 2006). How is cell membrane depolarization able to alter large‐scale anatomy? We identified several large‐scale‐function gene clusters within the frog dataset (Fig. 4B, Table 1). These included regulation of various stages of the cell cycle, cell survival, cell proliferation, cell differentiation, dedifferentiation, and cell death. Depolarization‐mediated regulation of such large‐scale functions was also found to be a common theme across all three (frog, axolotl, and human) datasets (Table 1). These data suggest that depolarization regulates development of tissues and organ/organ systems across all germ layers, perhaps by controlling such basic large‐scale functions found across all cells and tissues.

The rhythmic oscillation of *V*_mem_ throughout the cell cycle (Bregestovski et al. 1992) occurs in concert with the regulation of cell cycle and proliferation by stable resting potential in a range of embryonic, somatic, and neoplastic cells (Blackiston et al. 2009; McCaig et al. 2009; Sundelacruz et al. 2009; Lobikin et al. 2012; Adams & Levin 2013; Chernet & Levin 2013b, 2014). In this paper we identified cell cycle genes like *fos*, *p27*, and *mapk*, which were previously shown to be regulated by *V*_mem_ (Kong et al. 1991; Wang et al. 2003), and we also identified novel links between *V*_mem_ and some classic cell cycle regulatory genes such as *tp53* and *Ras*. These last two are particularly interesting because they suggest the presence of feedback loops in the control of oncogene‐driven tumorigenesis by depolarization (Chernet & Levin 2013a, 2013b, 2014).

In addition to being potent drivers of cell differentiation along various lineages (forming several different kinds of cells) (Ghiani et al. 1999; Bauer & Schwarz 2001; Chittajallu et al. 2002; Sundelacruz et al. 2008), *V*_mem_ signals also possess the capacity to induce dedifferentiation and transdifferentiation (Cone & Tongier 1971; Harrington & Becker 1973; Stillwell et al. 1973; Cone & Cone 1976; Sundelacruz et al. 2008, 2013a). In vivo experiments have shown the remarkable power of *V*_mem_ signals to re‐specify organ identity by inducing cell differentiation across germ layers (Beane et al. 2011, 2013; Pai et al. 2012a, 2015). Here we identified for the first time fate‐specification genes regulated by *V*_mem_ for tissues from all three germ layers (Table 1 and Appendix S2) (e.g., *nkx2.5*, *gata4*, *nkx3.1*, *hnf1b*) (Tronche & Yaniv 1992; Shiojima et al. 1995; Bhatia‐Gaur et al. 1999; Zhu et al. 2000; Kohler et al. 2008; Li et al. 2012; Zhou et al. 2012). These data suggest that other organs such as heart, liver, kidney, pancreas, bone fat, muscles, and immune system may also be inducible by appropriate *V*_mem_ modulation, in addition to the ectopic brains, eyes, and limbs that have been produced so far.

Programmed cell death or apoptosis mediates tissue morphogenesis during development and is in fact required for regeneration (Schwartz 1991; Abud 2004; Tseng et al. 2007; Li et al. 2010; Ryoo & Bergmann 2012). Recent studies have shown *V*_mem_ regulation of apoptosis as a way of regulating morphogenesis and tissue remodeling (Lang et al. 2005; Beane et al. 2013; Englund et al. 2014). But how *V*_mem_ regulates apoptosis is not well understood. Our analysis not only identified putative transcriptional targets by which *V*_mem_ regulates apoptosis (Table 1 and Fig. 4), but also identified other cell death mechanisms such as anoikis that are regulated by *V*_mem_ (Table 1). It offers valuable insight into mechanisms of cell death regulation by indicating mitochondrial membrane potential and genes such as *bax* as targets of *V*_mem_ signals (Table 1 and Appendix S2). The harnessing of these endpoints by targeted bioelectric modulation, using currently available technologies such as pharmacological cocktails (Tseng et al. 2010; Famm et al. 2013; Sinha 2013), optogenetics (Bernstein et al. 2012; Adams et al. 2013; Spencer Adams et al. 2014), and gene therapy (Chernet & Levin 2013b, 2014; Pai et al. 2015), is an exciting area for the extension of synthetic bioengineering techniques.

### Depolarization regulates developmental signals across diverse species

A common subset of cellular‐level signaling pathways responsive to depolarization was found to be differentially affected in all three datasets (Table S2). The majority of these pathways belonged to the “developmental simaton” (defined here for the first time as a conserved collection of juxtacrine and paracrine signals for growth factors, morphogens, hormones, and cytokines known to integrate together in various spatial and temporal permutations−combinations to drive specific developmental patterns) (Table S2). SNEA for “expression targets” in Pathway Studio showed that BMP2 was regulated in the frog, axolotl, and human cell experiments (pre‐differentiation) (Figs. 5A, S2A and Appendix S3). BMP2 was also identified as being enriched as a regulator of downstream expression targets that was also differentially regulated in the dataset (i.e., gene hub). Our findings support recent work showing BMP expression to be downstream of ion channel function (Dahal et al. 2012; Swapna & Borodinsky 2012). Strikingly, a deeper analysis of the *Xenopus* array revealed that the developmental simaton (conserved across species) pathways such as insulin‐like growth factor receptor 1 (*igfr1*), fibroblast growth factor receptor 3 (*fgfr3*), and others are involved in depolarization‐regulated tissue/organ development processes across all three germ layers (brain/neural, skeletal/bone, adipose, and immune system), supporting their role as a master‐regulator for specific organs/organ systems (Tseng & Levin 2013a; Levin 2014a). Here we have analyzed only a single snapshot of the interaction of *V*_mem_ with the developmental simaton. A temporal analysis is required for revealing further dynamic interactions between *V*_mem_ and components of the developmental simaton.

Another set of depolarization‐regulated signals conserved across species (frog, axolotl, and human datasets) are ion translocators, particularly those mediating calcium and chloride transport and signaling (Figs. 5B, S2B, C). Previous studies have shown that calcium signaling is an important transduction mechanism for *V*_mem_‐mediated regulation of brain and eye patterning (Beane et al. 2011; Pai et al. 2012a). Interestingly, there appears to be crosstalk between the developmental simaton signals like BMP2 and BDNF and the ion‐translocator‐mediated signals (Figs. 5B, S2B, C). The regulation of calcium and chloride transport and signaling, and their crosstalk with the developmental simaton signals, suggests the presence of feedback loops, where the bioelectric state change regulates (directly or indirectly via its influence on the developmental simaton) expression of new ion channels which in turn further alter the bioelectric landscape an ongoing cycle of feedback between physiological and transcriptional dynamics.

Another mechanism downstream of voltage change is bioelectric regulation of serotonergic signaling, which has been characterized in the role of *V*_mem_ in left−right patterning, neural pathfinding, and melanoma‐like transformation (Levin et al. 2006; Blackiston et al. 2011, 2015; Lobikin et al. 2012). Our analysis indicated additional neurotransmitters which have not yet been investigated in this context (Table 3 and Fig. S3). One example is *slc1a3* the sodium‐dependent glutamate/aspartate transporter which suggests that these neurotransmitters should be tested in developmental bioelectricity assays. Similar observations have also been made with respect to the dopamine system in *Xenopus* development (Langlois & Martyniuk 2013).

Overall, the data indicate that there are certain signaling pathways induced by depolarization that seem to regulate processes at all levels of organization cellular, tissue, organ, and organ system levels. Specifically, depolarization regulates cell cycle pathways (oncogenic/tumor suppressor) which control large‐scale (basic broad spectrum) functions like cell fate, dedifferentiation, proliferation, and death. It can be hypothesized that *V*_mem_ control of such basic large‐scale function, in turn, may allow it to regulate development of multiple tissues and organs across all germ layers during embryonic development. This is supported by the documented role of bioelectricity as a master‐regulator for specific organs/organ systems (Tseng & Levin 2013a; Levin 2014a). Our data suggest that bioelectric regulation of developmental simatons may be one of the mechanisms by which ionic signaling exerts long‐range, non‐cell‐autonomous effects not only during developmental patterning (Vandenberg et al. 2011; Pai et al. 2012a, 2012b, 2015) but also in tumor suppression (Chernet & Levin 2014), metastatic induction (Blackiston et al. 2011; Lobikin et al. 2012), and regenerative remodeling (Oviedo et al. 2010; Beane et al. 2011, 2012, 2013; Lobo et al. 2012). Most importantly, the various targets of depolarization are found to be largely conserved themes among three very diverse model systems (frog, axolotl, and human) and distinct in vivo and in vitro contexts (development, regeneration, and stem cell biology) analyzed. This suggests a conserved set of responses that will facilitate the medical exploitation of bioelectric control methods.

### Depolarization may regulate gene networks related to human disease

Based on gene expression profiles, our data revealed that oncogene/tumor suppressor genes are the second largest group of pathways regulated by depolarization (Tables 3 and S2), providing a mechanistic link between the known involvement of ion channels in cancer (Kunzelmann 2005; Pardo et al. 2005; Felipe et al. 2006; Stuhmer et al. 2006; Prevarskaya et al. 2010; Lobikin et al. 2012; Yang & Brackenbury 2013; Lang & Stournaras 2014). To determine which other diseases might be associated with depolarization, we identified the disease networks associated with differential gene expression in all three (frog, axolotl, and human) datasets. As expected, the largest group of diseases were neoplasms associated with various tissues (Table 2). Numerous studies have now shown an intimate relationship between ion channels and human cancers, and targeting ion channels is now being considered as a novel therapy for personalized non‐genetic cancer treatment (Schonherr 2005; Fiske et al. 2006; Arcangeli et al. 2009; House et al. 2010; Lobikin et al. 2012).

Surprisingly, a significant number of metabolic disease networks like diabetes, insulin resistance, and glucose intolerance were also related to genes regulated by depolarization (Fig. 6A and Table 2). Other disease networks include those related to neural diseases, immune disorders, cardiac disorders, pulmonary disorders, developmental disorders/birth defects, and wound healing and regenerative defects (Table 2). We observed an underlying theme of hypertrophic diseases across tissues in relation to depolarization, for example cardiac hypertrophy, cardiomegaly, pulmonary fibrosis, pulmonary hypertension, and even inflammation and arthritis (Table 3). This is probably due to the regulation of basic cell processes like cell proliferation, differentiation, and death by depolarization. While a range of channelopathies reveal ion channels at the root of birth defects (see Table 1 in Levin [2013]), most of these have not been specifically linked to depolarization. Thus, our data suggest specific clinical endpoints that should also be investigated with respect to resting potential change. Our long‐term goal is to apply this knowledge to develop bioelectric strategies to address disease states for regenerative medicine.

The neural disorders, especially neurotoxicity and nerve degeneration (Figs. 6B and S3), are particularly interesting as we found neurotransmitter pathways (Table 3) and neurodegenerative pathways (e.g., Parkinson's and Huntington's) (Table 3 and Fig. S3) to be induced by depolarization, suggesting that resting potential changes may represent a functional therapeutic target in these cases. Experiments in *Xenopus* have shown that enforcing correct nervous system *V*_mem_ signals can rectify developmental brain defects (Pai et al. 2012a, 2015). Such information is valuable because a large panel of ion channel drugs exists, many already approved for human use, and can be tapped as off‐label uses for electroceuticals in the nervous system and non‐neural tissues. Another disease group of note is the wound healing and regeneration defects, providing gene targets as hypotheses to be tested during *V*_mem_’s known effects on wound healing and regenerative processes (Chifflet et al. 2005; Cao et al. 2011a, 2011b; Vieira et al. 2011; Luxardi et al. 2014).

### Conclusions

**Figure 7:**
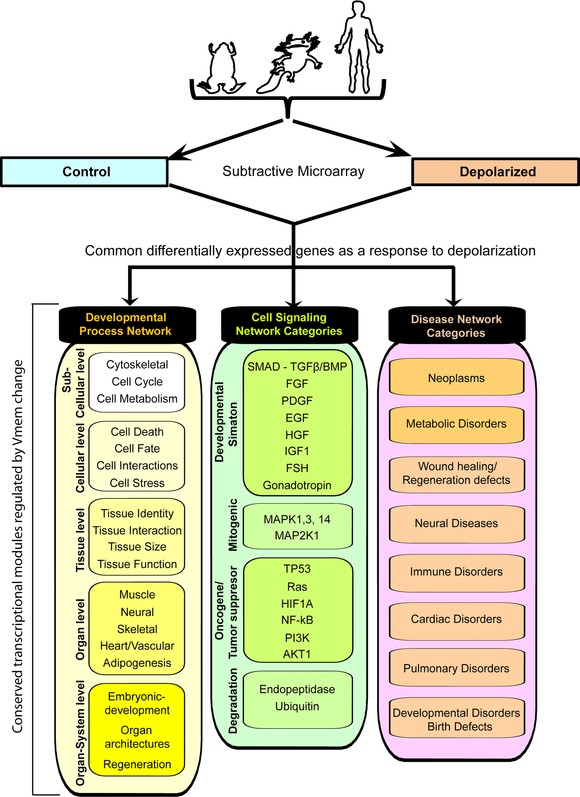
Summary of comparative genomic analysis, showing common themes of differentially expressed genes in response to depolarization.

A large number of cell processes that are regulated by *V*_mem_ change were found to be in common among amphibian and human systems, and across contexts of embryogenesis, spinal cord regeneration, and adult stem cells (Fig. 7). Our data are consistent with a highly conserved role of bioelectric state as a major regulator of cell activity and pattern regulation at all levels of organization cellular, tissue, organ, and whole body axes, both in vitro and in vivo. The consequences of depolarization were observed in transcripts characteristic of all germ layers (ectoderm, mesoderm, endoderm, and neural crest), highlighting resting potential as a powerful control point for biomedical interventions. The identification of neurotransmitter pathways linked to voltage change in somatic cells suggests not only new models of signal transduction but also the possible relevance of models of synaptic information processing that may be relevant to cellular decision‐making during pattern regulation. The identification of disease pathways suggests that bioelectric signaling and intervention using electroceutical strategies should be examined for diseases such as cancers of several different tissues, metabolic disorders like diabetes, congenital malformations (e.g., neural tube defects), and neurodegenerative diseases. Taken together, our data provide a framework for testing mechanistic hypotheses for bioelectric pathways, as novel components for synthetic bioengineering circuits using ionic signaling, and as targets for biomedical intervention in a range of disease states.

## Methods

### *Xenopus laevis* (Frog)

#### Animal husbandry

*Xenopus laevis* embryos were fertilized in vitro according to standard protocols (Sive et al. 2000) in 0.1× Marc's modified Ringer's solution (10 mmol/L Na^+^, 0.2 mmol/L K^+^, 10.5 mmol/L Cl^−^, 0.2 mmol/L Ca^2+^, pH 7.8). Intracellular ion concentrations in *Xenopus* embryos are 21 mmol/L Na^+^, 90 mmol/L K^+^, 60 mmol/L Cl^−^, 0.5 mmol/L Ca^2+^ (Gillespie 1983). *Xenopus* embryos were housed at 14–18°C and staged according to Nieuwkoop and Faber (1967). All experiments were approved by the Tufts University Animal Research Committee (M2014‐79) in accordance with the guide for care and use of laboratory animals.

#### Microinjections

Capped synthetic mRNAs generated using mMessage mMachine kit (Ambion, ThermoFisher Scientific, Grand Island, NY, USA) were dissolved in nuclease‐free water and injected into the embryos in 3% Ficoll using standard methods (Sive et al. 2000). Each injection delivered between 1 and 2 nL or 1 and 2 ng of mRNA (per blastomere) into the embryos, usually at the one‐cell stage into the middle of the cell in the animal pole. Constructs used were GlyR (Davies et al. 2003) and 666 chimera (Hough et al. 2000).

#### RNA extraction and microarray analysis

The procedure was performed by the Beth Israel Deaconess Medical Center (BIDMC) Genomics Core (Boston, MA) at Harvard University. A miRNeasy Mini kit (Qiagen, Hilden, Germany) was used to isolate total RNA from the *Xenopus* embryos. There were 50 embryos pooled for each experimental sample. Microarray hybridization target preparation was performed using the Affy 3′IVT Express Kit (Affymetrix, Santa Clara, CA) as per the manufacturer's protocol. Fragmented and biotin labeled/amplified RNA was hybridized to the GeneChip® *Xenopus laevis* Genome 2.0 array (Affymetrix, Santa Clara, CA) as per the protocol provided by the manufacturer. The Affymetrix GeneChip® *X. laevis* Genome 2.0 Array has 32,400 probe sets representing more than 29,900 *X. laevis* transcripts. The quality of hybridized arrays was assessed using Affymetrix guidelines on the basis of scaling factor, background value, mean intensity of chip and 3′ to 5′ ratios for spike‐in control transcripts. The outlier analysis was performed using unsupervised clustering and principal components analysis. All high quality arrays were normalized using the MAS5 algorithm developed by Affymetrix. The absent/present calls for the transcripts were calculated using the MAS5 algorithm. The differentially expressed transcripts were identified on the basis of fold change and Affymetrix transcript calls. Overexpressed transcripts were at least twofold in the experimental group compared to the control group with present call in the experimental group. Under‐expressed transcripts were at least twofold changed in the experimental group compared to the control group with present call in the control group. Differentially expressed genes were annotated using the Affymetrix database or by performing BLAST analysis. All microarray data were analyzed using Bioconductor packages in R. The NCBI GEO accession number for frog data is GSE72099.

#### Experimental design rationale

Each (RNA) sample, including the control, was generated not from a single individual embryo but by pooling 50 embryos (including control). This gives a robust average expression compared to using single embryos, and smooths out any individual variability. In addition, we pursued a more nuanced and strict replication strategy than simple replicates; this was necessary here because of the specific nature of depolarization as a developmental signal. Traditional replicates would filter out technical noise but would still include genes specifically responsive to a given experimental technique (e.g., IVM exposure), as opposed to what we wanted to study: the effects of depolarization per se. We used two different depolarization methods/samples (each one with 50 embryos pooled together), and kept only those transcripts that were shown to be similarly regulated in both treatments. This new strategy not only filters out technical noise (as would traditional replicates), but also filters out transcripts that are not consistent between the two different ways of depolarization a much stronger criterion that removes a lot more noise, both technical variability and responses that are specific to a single type of perturbation. This provides a stringently curated set of targets related to what we wanted to study depolarization and comes from two methods/samples (each sample representing 50 embryos).

### *Ambystoma mexicanus* (Axolotls)

#### Animal husbandry

All axolotls used in these experiments were bred in the axolotl facility at the University of Minnesota under the IACUC protocol #1201A08381. Axolotls of 2–3 cm were used for all in vivo experiments, and animals were kept in separate containers and fed daily with artemia; water was changed daily. Animals were anesthetized in 0.01% *p*‐amino benzocaine (Sigma‐Aldrich, St. Louis, MO, USA) before microinjection was performed.

#### Experimental design: ivermectin injection

IVM or vehicle only (water) was pressure injected into the central canal of the spinal cord, and this was visualized by the addition of Fast Green into the solution. Directly after injection, a portion of the spinal cord was surgically removed and the animals were placed back into water in individual containers. One day post‐injury animals were anesthetized again and the area of the injury was removed. Tissue from 10 animals was pooled for each microarray replicate.

#### RNA extraction and microarray analysis

Total RNA extraction for microarray analysis was done using TRIzol® Reagent. RNA was resuspended in 20 μL of RNAase‐free water. RNA concentration was measured using a Nano‐Drop 2000 spectrophotometer (ThermoFisher Scientific, Grand Island, NY, USA). RNA integrity was evaluated using an Agilent 2100 Bioanalyser. Microarrays were carried out in triplicate using a custom‐made axolotl Affymetrix Chip, and arrays were processed at the microarray facility at the MPI‐CBG, Dresden, Germany. Briefly, for each sample, 150 ng total RNA was used to reverse transcribe double‐stranded cDNA and subsequently in vitro transcribe biotin‐labeled target cRNA as per the GeneChip 3′IVT Express Kit. The target cRNAs were hybridized to Amby002: a custom Affymetrix GeneChip©. The chip contains 20,000 axolotl probes. Hybridizations were also performed according to the GeneChip 3′IVT Express Kit as per the user manual. Approximately 12.5 μg fragmented and labeled aRNA was hybridized for 17 h at 45°C and 60 rpm. Arrays were washed and stained using the GeneChip Fluidic station FS450 with the wash and stain protocol FS450_0001. The arrays were scanned using the Affymetrix GCS3000 System (Scanner) using default parameters to obtain background‐corrected signal intensities. Cel files containing normalized intensity data (RMA normalization) were generated using the Gene Expression Console (Affymetrix). FDR was not used for the axolotl data. The NCBI GEO accession number for axolotl data is GSE72099.

### Human mesenchymal stem cells (hMSCs)

#### Experimental design: hMSC cultivation

Whole bone marrow aspirate from a 25‐year‐old healthy man was purchased from Lonza through their Research Bone Marrow Donor Program, following approved guidelines of informed consent as previously documented (Sundelacruz et al. 2008, 2013a). Aspirate was plated at a density of 10 mL of aspirate per square centimeter in control medium (Dulbecco's modified Eagle's medium with 10% fetal bovine serum, penicillin [100 U/mL], streptomycin [100 mg/mL], and 0.1 mmol/L nonessential amino acids) supplemented with basic fibroblast growth factor (1 ng/mL) (Invitrogen, ThermoFisher Scientific, Grand Island, NY, USA). Cells were maintained in a humidified incubator at 37°C with 5% CO_2_. The hMSCs were isolated on the basis of their adherence to tissue culture plastic and were used for experiments between passages two and four.

#### Differentiation of hMSCs

Undifferentiated hMSCs were cultured in control medium. Osteogenic differentiation medium consisted of α‐modified minimum essential medium supplemented with 10% fetal bovine serum, penicillin (100 U/mL), streptomycin (100 mg/mL), 10 mmol/L β‐glycerophosphate, 0.05 mmol/L l‐ascorbic acid‐2‐phosphate, and 100 nmol/L dexamethasone (Sigma‐Aldrich).

#### Depolarization of membrane potential

*V*_mem_ was depolarized by (1) addition of OB (10 nmol/L; Sigma‐Aldrich) to differentiation medium or (2) elevation of extracellular K^+^ by adding potassium gluconate (40 mmol/L; Sigma‐Aldrich) to differentiation medium. Depolarization induced by these concentrations of OB and K^+^ has been confirmed using voltage‐sensitive dyes and/or sharp intracellular recordings (Sundelacruz et al. 2008; Kaplan DL et al. unpublished data). Osteogenic cells were pre‐differentiated for 3 weeks before the addition of OB or potassium gluconate for an additional 3 weeks of culture. The depolarizers were added to the differentiation medium and replenished in subsequent media changes.

#### RNA extraction and microarray analysis

RNA was isolated from samples collected in TRIzol® reagent according to the single‐step guanidinium acid‐phenol method. RNA was further purified using the RNeasy Mini kit according to the manufacturer's instructions, which included an on‐column DNase treatment. RNA integrity was evaluated with the Agilent Bioanalyzer. The cDNA reverse transcription, cDNA purification, in vitro transcription of cRNA, and microarray hybridization, staining, and scanning were performed by the Yale Center for Genomic Analysis. We used Illumina Human WG6 v3 Expression BeadChip arrays, which have 48,804 probe sets, of which over 27,000 represent coding transcripts with well‐established annotation. Sample sizes were as follows: depolarized groups, *n* = 3; non‐depolarized osteogenic control group, *n* = 3; undifferentiated control group, *n* = 2.

Data analysis was performed using Bioconductor software packages. The *lumi* package (Du et al. 2008; Lin et al. 2008) was used to perform variance stabilizing transformation and quantile normalization, and the *limma* package (Smyth 2004) was used for linear modeling and differential expression analysis. Log2‐fold gene expression changes and empirical Bayes moderated *t* statistics were computed, with *P* values adjusted according to the Benjamini−Hochberg method and considered significant for *P* < 0.01. We performed hypergeometric testing on Gene Ontology terms using the *GOstats* package (Gentleman 2004) and adjusted *P* values for multiple testing, considering results significant for *P* < 0.01. The NCBI GEO accession number for hMSCs data is GSE72099.

### Bioinformatics

#### Subnetwork enrichment and pathway analysis

SNEA was performed in Pathway Studio 9.0 (Elsevier Life Science Solutions) and ResNet 9.0 to construct gene interaction networks for transcripts showing differential expression in frog, axolotl, and human cells. For each of the three datasets, the list of differentially expressed genes was mapped into the program using official gene symbols (Name + Alias). SNEA was performed and significantly enriched processes were determined to be those with *P* < 0.05 that also contained more than 10 members in the network. These were the networks that are most represented by the entities in each gene list. Both cell processes and disease subnetworks were queried and there were 500 permutations of the data to generate the distributions. Briefly, SNEA uses known relationships among genes (e.g., relationships based on co‐expression patterns, binding, or involvement in common pathways) to build networks focused around gene hubs. These interaction maps are generated using information from the ResNet 9 database. The database contains over 20 million PubMed abstracts and ∼2.4 million full‐text articles (22 September 2014). Thus, these are pre‐defined molecular networks based on the literature (i.e., it is the background or reference group). A gene list is then imported into the program and a statistical comparison between the experimental subnetworks mapped to known networks and the entire background of known networks (reference group) is conducted using a Mann−Whitney *U* test; a *P* value is generated that indicates the statistical significance of difference between two distributions (additional details on the method can be found in the technical bulletin, page 717, from Pathway Studios 7.0). Venn diagrams (Oliveros 2007) were generated using both official gene symbols and pathways to identify common genes and pathways affected in all three experiments.

Gene networks based upon expression, binding, and regulatory interactions of entities were constructed using direct connections with one neighbor. In order to simplify the pathways, only those interactions with the highest scores (most well supported by the literature) are shown (>3000 connectivity; local connectivity >5). However, it is important to note that the pathways are built with additional information that is not shown in the figures (i.e., those connections showing <3000 connectivity). Subnetworks that included “cell process,” “expression targets,” and “disease” were queried for all three species. We reasoned that those processes in common among the datasets were those most responsive to depolarization. These data were then subjected to manual categorization into “major biological themes.”

#### PANTHER analysis

The gene lists from the microarray experiments were uploaded to the PANTHER database and functional classification was viewed as pie charts as previously documented (Thomas et al. 2006; Mi et al. 2013a, 2013b).

## Supporting information

Additional Supporting Information may be found in the online version of this article at the publisher's website:

**Figure S1**. Subnetwork enrichment analysis of *Xenopus* dataset identified (A) regulated genes that are involved in adipocyte differentiation and (B) regulated genes that are involved in the immune system. Acronyms can be found in Appendix S5. Gene functions can be found in Table S1.
**Figure S2**. (A) Subnetwork enrichment analysis of the human database identifies regulated genes that are involved in BMP2 signaling. Acronyms can be found in Appendix S4. (B) Subnetwork enrichment analysis of the axolotl database identifies regulated genes that are involved in calcium signaling. Acronyms can be found in Appendix S5. (C) Subnetwork enrichment analysis of the human dataset identifies regulated genes that are involved in calcium signaling. Acronyms can be found in Appendix S5. (D) Subnetwork enrichment analysis of the *Xenopus* database identifies regulated genes that are involved in chloride transport. Acronyms can be found in Appendix S5.
**Figure S3**. Subnetwork enrichment analysis of the axolotl database identifies (A) regulated genes involved in Huntington disease pathway and (B) regulated genes involved in Parkinson disease pathway. Acronyms can be found in Appendix S5.
**Table S1**. List of genes from subnetwork enrichment analysis of *Xenopus* genes involved in organogenesis.
**Table S2**. List of cell signaling pathways from subnetwork enrichment analysis that are common to all three (frog, axolotl, and human) datasets.
**Appendix 1**. Entire list of differentially expressed genes in response to depolarization from all three species, frog, axolotl, and human.
**Appendix 2**. Entire list of enriched cell processes in response to depolarization from all three species, frog, axolotl, and human.
**Appendix 3**. Entire list of enriched expression targets in response to depolarization from all three species, frog, axolotl, and human.
**Appendix 4**. Entire list of enriched disease networks in response to depolarization from all three species, frog, axolotl, and human.
**Appendix 5**. Entire list of gene acronyms used in the depiction of gene networks in the figures.
