# Supplementary material for: Genome‐wide analysis reveals conserved transcriptional responses downstream of resting potential change in Xenopus embryos, axolotl regeneration, and human mesenchymal cell differentiation
Source: Regeneration (Oxf). 2015 Nov 26;3(1):3–25. doi: 10.1002/reg2.48 (PMC4857752; doi:10.1002/reg2.48)
Supplement: Supplementary file 1 — Figure S1. Subnetwork enrichment analysis of Xenopus dataset identified (A) regulated genes that are involved in adipocyte differentiation and (B) regulated genes that are involved in the immune system. Acronyms can be found in Appendix S5. Gene functions can be found in Table S1. Figure S2. (A) Subnetwork enrichment analysis of the human database identifies regulated genes that are involved in BMP2 signaling. Acronyms can be found in Appendix S4. (B) Subnetwork enrichment analysis of the axolotl database identifies regulated genes that are involved in calcium signaling. Acronyms can be found in Appendix S5. (C) Subnetwork enrichment analysis of the human dataset identifies regulated genes that are involved in calcium signaling. Acronyms can be found in Appendix S5. (D) Subnetwork enrichment analysis of the Xenopus database identifies regulated genes that are involved in chloride transport. Acronyms can be found in Appendix S5. Figure S3. Subnetwork enrichment analysis of the axolotl database identifies (A) regulated genes involved in Huntington disease pathway and (B) regulated genes involved in Parkinson disease pathway. Acronyms can be found in Appendix S5. Table S1. List of genes from subnetwork enrichment analysis of Xenopus genes involved in organogenesis. Table S2. List of cell signaling pathways from subnetwork enrichment analysis that are common to all three (frog, axolotl, and human) datasets. Appendix 1. Entire list of differentially expressed genes in response to depolarization from all three species, frog, axolotl, and human. Appendix 2. Entire list of enriched cell processes in response to depolarization from all three species, frog, axolotl, and human. Appendix 3. Entire list of enriched expression targets in response to depolarization from all three species, frog, axolotl, and human. Appendix 4. Entire list of enriched disease networks in response to depolarization from all three species, frog, axolotl, and human. Appendix 5. Entire list of gene acro [file REG2-3-03-s001.zip › Supplemental Table 2 - SNEA - Cell signaling targets common to all 3 datasets.docx]

Supplemental Table 2 – Common cell signaling pathways in all 3 (frog, axolotl and human) datasets

| ***Genes*** | ***Pathway category*** |
| --- | --- |
| proteasome endopeptidase complex | Degradation Pathways |
| ubiquitin |  |
| TP53 - (tumor protein p53) | Oncogene/Tumor suppressor |
| Ras |  |
| HIF1A |  |
| NF-kB |  |
| PI3K |  |
| AKT1 |  |
| MAPK14 | Mitogenic pathways |
| MAP2K1 |  |
| MAPK1 |  |
| MAPK3 |  |
| SMAD (TGFβ/BMP pathway) | Developmental Simaton Pathway |
| SMAD3 (TGFβ/BMP pathway) |  |
| FGF2 |  |
| FGF |  |
| PDGF |  |
| EGF |  |
| EGFR |  |
| HGF |  |
| gonadotropin |  |
| FSH |  |
| IGF1 |  |
